# Periscope: Surgery

**Published:** 1818-10-01

**Authors:** 


### Surgery.

1. *Extensive Abscesses.* Mr. Cunningham, Surgeon in the Royal Navy, draws the attention of the chirurgical practitioner *(Med. Chir. Journ. April)* towards an improved method of treating extensive suppurations; namely, to lay the sac of the abscess open through its whole extent with a bistoury, press out the matter, bring the edges of the incision into contact, and, by means of compresses and bandages, with evaporating lotions, induce a union, without further secretion of pus, between the parietes of the abscess. Several cases are related where this plan was successfully practised; and the writer of this Periscope saw one example, where, he is confident, the common mode of evacuating the suppurated collection would have required weeks or months for a cure, which, on Mr. C's plan, was effected in four days.

2. *Treatment of Phlegmon.* Mr. Drapes, of Ross, contends *(Ed. Journ. 54)*, that in true phlegmon, as known by "circumscribed tumour, with heat, redness, tension, throbbing pain, &c." resolution *never* takes place, and consequently that all cold or evaporating lotions are not only useless but injurious. He therefore recommends fomentations and poultices, in all stages of the tumour, till it breaks or is opened. Every man of experience must have seen so many instances of resolution in phlegmon, that the foregoing dogma must appear an insult to his understanding.

3. *Wounds of the Heart.* Two instances of this kind are related in the 54th number of the *Ed. Journal*; one by Mr. John Fuge, of Plymouth, the other by the Editor. The first was a soldier, who received a musket shot in the chest, in Spain, the evening before the battle of Corunna. The ball penetrated the right ventricle, and recoiled back into the cavity of the pericardium, where it was found. He lived nearly fourteen days. The second case happened in London, and the man lived about forty-four hours. The ball had penetrated the right ventricle also, and recoiled back into the pericardium.

4. *Treatment of Syphilis without Mercury.* Deputy Inspector Hennen, in the same Journal, states the great success that has attended the treatment of all forms of syphilis, primary and secondary, without mercury, in the military hospitals in Edinburgh. Much as we respect the opinions of Mr. Hennen, we hesitate not to stake our prognostic characters, that the whole business will turn out, in the end, a self-deception, notwithstanding the wisdom which directs, and the talent which conducts this important investigation.' In the succeeding number of the same Journal, Mr. Hennen returns to the charge.

The following sentence is not very encouraging to the non-mercurial practice.

"The facts at present ascertained are these:—secondary symptoms occur more frequently, and appear at an earlier and more determinate period than when mercury has been used." P. 332.

Mr. Alcock (*Repos.* 54) a very intelligent and well informed surgeon in Piccadilly, has stated some interesting facts relative to the early destruction of chancrous sores on the penis by escharotics, thereby rendering unnecessary the employment of mercury. During the last ten years he has frequently used this practice, and had not once occasion to regret its adoption. He generally gives the preference to a *saturated* solution of sulphate of copper, which causes less pain than lunar caustic.

"The chancre having been washed with warm water, and wiped dry, I immediately apply the solution by a camel hair pencil, or other convenient medium, to the whole surface of the sore, carefully observing that every point of the latter be included, and continuing the application of the solution from one to two minutes, till an eschar be fully formed."

Only dry lint or simple dressing is afterwards applied; and if the eschar do not drop off by the second day, it is removed by the probe, forceps, or scissors, and the same application is again renewed. After the second or third process of this kind, the sore generally loses its specific character, and heals kindly. For various interesting remarks on the subject, we must refer to the paper itself, which evinces a mind in the author at once original, comprehensive, and intelligent.

5. *Gonorrhœa treated various ways.* Messrs. Johnston and Bartlett, of the 88th Regiment, have made trials of different modes of cure in this complaint. 1st. A solution of lunar caustic in plain boiled water (twenty grains to the ounce) was tried as injection, and the average time of cure was seventeen days. 2d. Rest and abstinence cured them, on an average, in eight days and a half. 3d. The piper cubeba cured them in five days and a quarter. 4th. Capsicum in thirteen days and a half. 5th. Camphor in nine days.

From some little experience in these matters, we can assert that all irritating injections lay the foundation for strictures, and that the best method of cure is rest, abstinence, and diluting drinks, with plenty of gum arabic,

Hyoscyamus and camphor, when the chordee is troublesome, we have found the best anodyne.

When a gleet *only* remains, then the bals. copaiba is an excellent medicine. Indeed, we have lately ventured on its use where inflammation actually existed, with marked benefit.

6. *Lues Venerea.* Dr. Henry Robertson, of Conduit Street, (Med. Repos. 54) gives his opinion, derived from eighteen years experience in the army, that mercury, *moderately administered,* is the only effectual remedy, both in syphilis, and what is termed pseudo-syphilis. He says, that in eighteen years he never met with an instance of secondary symptoms where he had the management of the case *ab origine.* This is a strong expression, and such as few of his brethren can make.

7. *Taliacotian Art.* Mr. A. Copland Hutchinson, late Surgeon of the Royal Hospital, Deal, and a zealous, able, and active cultivator of operative and medical surgery, has lately performed the nasal operation, according to the plan recommended by Mr. Carpue, and with success. The patient, a female, had lost every part of the nose, even the nasal bones, about eight years ago, from gangrene succeeding an attack of erysipelas. It was not necessary to tie any blood-vessel. On the second day, a slight hæmorrhage occurred from the internal surface of the new nose. On the eleventh day from the operation, there was external hæmorrhage, to the amount of thirty-three ounces from the right angular artery, but which stopt spontaneously. The blood issued from the very part whence the stitch had been removed, and must have been caused by the ulceration of the coats of the vessel.

8. *Temporal Arteriotomy.* Mr. Ch. Kane *(Edinburgh Journal)* recommends, that in ophthalmia, where temporal arteriotomy is, he thinks, far superior to any other mode of depletion, the artery should be cut down upon and opened partially. After a sufficient quantity of blood has been abstracted, the vessel is to be entirely divided, and then he directs "to tie its main portion, cut both ends of the ligature *close* to the knot, and heal the wound by the first intention." Whatever improvement there may be in this practice, surely Mr. K. has greatly exaggerated the evil consequences of applying a compress to the wound of the temporal artery—"which," says he, "is a process *as baneful to the cause of science,* as destructive to the patient." P. 346.

